# Functional neural changes associated with acquired amusia across different stages of recovery after stroke

**DOI:** 10.1038/s41598-017-11841-6

**Published:** 2017-09-12

**Authors:** Aleksi J. Sihvonen, Teppo Särkämö, Pablo Ripollés, Vera Leo, Jani Saunavaara, Riitta Parkkola, Antoni Rodríguez-Fornells, Seppo Soinila

**Affiliations:** 1 Faculty of Medicine, University of Turku, 20520 Turku, Finland; 2 Cognitive Brain Research Unit, Department of Psychology and Logopedics, Faculty of Medicine, University of Helsinki, 00014 Helsinki, Finland; 3 Cognition and Brain Plasticity Group, Bellvitge Biomedical Research Institute (IDIBELL), L’Hospitalet de Llobregat, 08907 Barcelona, Spain; 4 Department of Cognition, Development and Education Psychology, University of Barcelona, 08035 Barcelona, Spain; 5 Poeppel Lab, Department of Psychology, New York University, 10003 NY, USA; 6 Department of Medical Physics, Turku University Hospital, 20521 Turku, Finland; 7 Department of Radiology, Turku University and Turku University Hospital, 20521 Turku, Finland; 8 Catalan Institution for Research and Advanced Studies, ICREA, Barcelona, Spain; 9 Division of Clinical Neurosciences, Turku University Hospital and Department of Neurology, University of Turku, 20521 Turku, Finland

## Abstract

Brain damage causing acquired amusia disrupts the functional music processing system, creating a unique opportunity to investigate the critical neural architectures of musical processing in the brain. In this longitudinal fMRI study of stroke patients (N = 41) with a 6-month follow-up, we used natural vocal music (sung with lyrics) and instrumental music stimuli to uncover brain activation and functional network connectivity changes associated with acquired amusia and its recovery. In the acute stage, amusic patients exhibited decreased activation in right superior temporal areas compared to non-amusic patients during instrumental music listening. During the follow-up, the activation deficits expanded to comprise a wide-spread bilateral frontal, temporal, and parietal network. The amusics showed less activation deficits to vocal music, suggesting preserved processing of singing in the amusic brain. Compared to non-recovered amusics, recovered amusics showed increased activation to instrumental music in bilateral frontoparietal areas at 3 months and in right middle and inferior frontal areas at 6 months. Amusia recovery was also associated with increased functional connectivity in right and left frontoparietal attention networks to instrumental music. Overall, our findings reveal the dynamic nature of deficient activation and connectivity patterns in acquired amusia and highlight the role of dorsal networks in amusia recovery.

## Introduction

During the past decades, the pursuit to unravel the neural structures underlying music processing in the brain has been very active. Modern neuroimaging methods evaluating both structure and function have provided evidence of a wide-spread music network in the healthy brain which comprises bilateral temporal, frontal, parietal, and subcortical regions^1–6^. While the ability to perceive and enjoy music is fundamental to humans across all cultures, the previously intact capability to perceive music can be impaired by brain damage (acquired amusia). In amusia, the deficit in processing pitch is considered as the signature symptom, but other domains of music, such as rhythm, timbre, memory, and emotions, can also be affected^7^.

Although one to two thirds of stroke patients have been reported to suffer from acquired amusia^8–10^, its neural basis has been previously poorly understood as previous studies have been limited to symptom-led and lesion-led studies of individual cases or small patient groups. Moreover, the results regarding the spatial location of the lesion, lesion lateralization (left, right), and the type of musical deficit (e.g. spectral, temporal) have been mixed. While acquired musical deficits have been associated with right hemisphere damage^11–17^, acquired amusia after both left and right hemisphere damage has been reported^8, 10, 18–20^. We recently showed, using voxel-based lesion-symptom mapping (VLSM), that acquired amusia after stroke is related specifically to damage in the right Heschl’s gyrus (HG), superior temporal gyrus (STG), and middle temporal gyrus (MTG) as well as the right insula and putamen^21^. Our findings have been supported by a recent study reporting musical short-term memory deficits in association with insular stroke lesions^22^. In our previous study, we also analyzed longitudinal grey and white matter changes associated with persistent acquired amusia using voxel-based morphometry (VBM). The VBM analysis revealed that non-recovering acquired amusia was associated with grey matter volume (GMV) decrease in the right STG and MTG as well as white matter (WM) volume decrease in MTG^21^. Moreover, we have recently replicated the VLSM and VBM results using a larger patient sample^23^.

However, while the findings described above provide crucial information about which damaged structures are linked to acquired amusia, they do not inform about the effect of acquired amusia on brain function or functional connectivity. Indeed, to our knowledge, no previous studies exist that had identified brain activity associated with music listening in acquired amusia. Moreover, the functional recovery and changes after acquired amusia has not been previously mapped. Traditionally, functional magnetic resonance imaging (fMRI) has been used to monitor recovery of function after stroke^24^. Overall, the recovery of function is supported by reorganization of the neural network and peri-infarct regions that survived the stroke^25^ and the greatest recovery is observed within the first months^26^. In post-stroke aphasia caused by left hemisphere damage, the recovery of language function has been observed to occur in three separate phases: (i) initial reduced activation in ipsilateral (left) frontotemporal language areas at acute stage, followed by (ii) upregulation or increase of activation in homologous contralesional (right) areas (especially right inferior frontal and supplementary motor areas) at subacute stage, and, finally, (iii) increased (normalized) activity in the ipsilateral (left) hemisphere at chronic stage^27^. This suggests that the brain activity patterns associated with the recovery can be dynamic across time, including the recruitment of different brain regions during acute and later phases of stroke, and overall, better language outcomes are correlated with increased fMRI activity^27^.

To date, there are only three published fMRI studies on amusia, all in congenital amusia^28–30^ and none in acquired amusia. Passive listening tasks with simple melodic sequence^28^ and harmonic tone^29^ stimuli have revealed normal activity patterns in the pitch-responsive brain regions in the auditory cortex (AC) in congenital amusics. However, the function of the right inferior frontal gyrus (IFG) as well as the connectivity between the right IFG and AC have been reported to be abnormal during a melody listening task^28^. In addition, using resting-state fMRI (i.e. without any musical stimulus), frontotemporal functional connectivity (FC) has been found to be reduced in congenital amusics^30^. However, while these studies have discovered neural structures involved in the processing of individual musical features in congenital amusia, they have utilized artificially manipulated and controlled auditory paradigms instead of naturalistic music. As music is much more than a sum of its acoustic components, studying natural music listening could provide more thorough and accurate outlook on how the processing of musical elements is affected in the amusic brain. In the healthy brain, fMRI studies utilizing natural music (real songs) have reported extensive activation patterns in a large-scale bilateral network of temporal, frontal, parietal, striatal, limbic, and cerebellar regions^4, 5^, compared to the more focal right frontotemporal activation underlying individual music-perceptual processes (i.e. pitch and rhythm perception). Logically, if the processing of musical components is affected in amusia, it should induce defective activation patterns in the large-scale music network when using natural music as stimuli.

Another core aspect in music processing is singing which combines characteristics from both language (e.g. syntax, semantics) and music (e.g. melody, harmony, rhythm). Evidence from neuroimaging studies show that there are both shared and distinct brain regions involved in singing and speaking: while both comprise a large network including sensorimotor areas and inferior frontal regions, compared to speaking, singing induces greater activations in the right STG and HG as well as the right pre- (PreCG) and postcentral gyrus (PCG) and the right IFG^31, 32^. Furthermore, to support the dissociation between language and music processing in the brain, both functions can be selectively impaired, suggesting, at least to a certain extent, separate neural correlates for both cognitive domains^33^. An interesting lateralization pattern is also present in vocal music: in addition to engaging the right STG and HG, music containing sung lyrics also activates left hemisphere brain areas associated with language processing, including the left STG and inferior temporal gyrus as well as the putamen, cuneus, PCG, and cerebellum^5^. Moreover, the lyrics and tunes in a song have been shown to have separate^34–37^ and integrated^38, 39^ processes in the brain. Interestingly, it has been suggested that congenital amusics can recognize the lyrics of familiar songs while they are unable to recognize the corresponding melodies^40^. Moreover, in congenital amusia, a singing intervention has been found to improve song production^41, 42^ and performance in the Scale^41^ and Meter^42^ subtests of the Montreal Battery of Evaluation of Amusia (MBEA). Congenital amusics can even reproduce pitch intervals in correct directions via singing while being unable to consciously perceive their differences^43^. Thus, the processing of vocal (sung) music may be partially spared in amusic subjects, but this has not been previously studied with fMRI.

Inferring possible functional deficits in acquired amusia on the basis of the published scarce evidence on congenital amusia cannot be done without careful consideration. While right frontotemporal structures and pathways are implicated in both congenital^44–47^ and acquired amusia^21–23^, the two types of amusia may have partly different neural bases. Congenital amusia is a life-long condition and therefore reflects not only impaired music perception, but also a developmental deficit in acquiring musical syntax and tonal representations^48^. Congenital amusia may also hinder exposure to music and hamper the development of the wide-spread music network in the brain^48^. In contrast, acquired amusia is characterized by a clear-cut shift from a normal to deficient function of the music processing system caused by a brain lesion. This provides an opportunity to examine the brain structures and functions that are crucial for music perception^49^.

To uncover the brain areas involved in music processing and also to unravel how the reorganization of the music network in acquired amusia takes place over time, fMRI detecting stimulus related brain activity can be used. Furthermore, fMRI can be utilized to assess the integrated activity of spatially distributed brain regions by evaluating FC. In this vein, one data driven approach to FC assessment is independent component analysis (ICA)^50^, which systematically explores temporally coherent brain regions (i.e. functional networks)^51^ without a priori expectations^52^.

In the present study, using 41 stroke patients, we utilized fMRI to uncover and differentiate activation patterns induced by natural music listening in acquired amusia for first time to our knowledge. Within 3 weeks of the stroke onset, all patients were evaluated for amusia and fMRI scans during the presentation (i.e. free spontaneous listening) of instrumental and vocal music stimuli (song excerpts either played instrumentally or sung with lyrics) were acquired. This procedure was repeated during the follow-up 3 and 6 months post-stroke. We also assessed brain activation changes associated with the recovery of amusia to determine, whether the recovery is due to canonical or additional compensatory brain areas. Analyses were performed both longitudinally and cross-sectionally in order to determine music-associated regions showing linear changes across time as well as explore the potentially dynamic nature of changing activation patterns at each time point, as has been observed in aphasia^27^. In addition, we used task-specific ICA to evaluate FC deficits associated with acquired amusia during music listening. Importantly, in contrast to previous studies using simple melodic tone sequences^28^ or harmonic tone complexes^29^, we utilized more ecologically valid natural musical stimuli comprising excerpts from well-known songs with or without lyrics. Based on the previously published literature on acquired amusia, our previous VLSM/VBM results, and on the few available fMRI studies published on congenital amusia, we hypothesized that (i) amusia is associated with decreased activations and FC in the right temporal and frontal areas, (ii) amusia recovery is due to perceived/increased activation in the right temporal areas, and (iii) activation patterns of vocal and instrumental music differ between amusic and non-amusic patients. In addition to the right frontotemporal regions that have been closely linked to amusia, both structurally and functionally, we also sought to explore whether also other brain networks (e.g. frontoparietal, limbic, striatal, cerebellar) would show changes in the processing of natural music during amusia recovery.

## Results

### fMRI activation patterns to music in all patients

**Figure 1:**
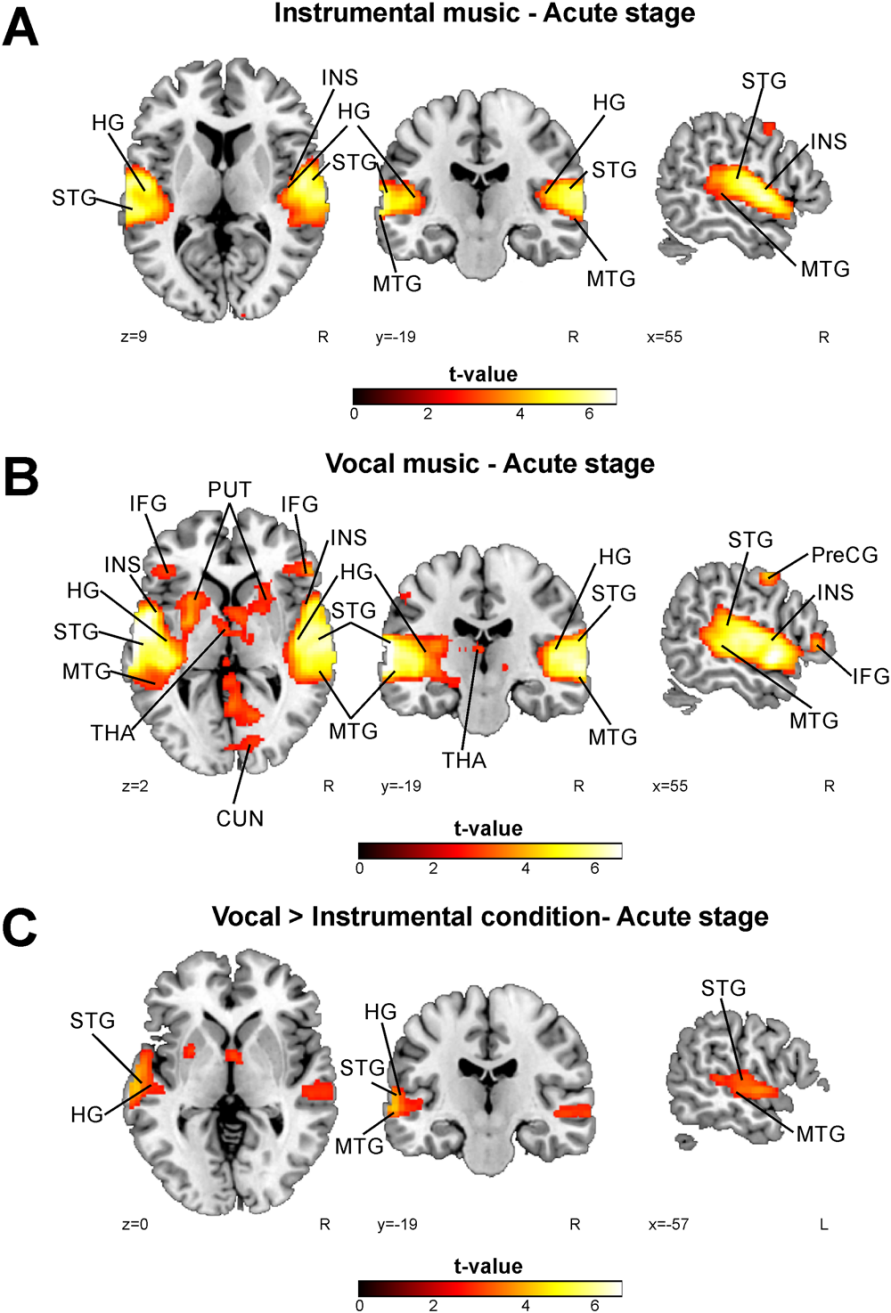
fMRI activations induced by instrumental and vocal music listening in the acute stage shown by one sample t-tests including all patients. (**A**) Instrumental music; (**B**) Vocal music; (**C**) Vocal music vs. instrumental music. N = 41. Results are shown at *p* < 0.005 (uncorrected) with ≥50 voxels of spatial extent and overlaid over a canonical template with MNI coordinates at the bottom right of each slice. Only clusters surviving a FWE-corrected *p* < 0.05 threshold are labeled (see also Table 1). HG = Heschl’s gyrus, IFG = inferior frontal gyrus, INS = insula, MTG = middle temporal gyrus, PreCG = precentral gyrus, PUT = putamen, SMA = supplementary motor area, STG = superior temporal gyrus, THA = thalamus.

To evaluate the activation patterns during each condition of interest (Vocal, Instrumental, Vocal > Instrumental), a one sample t-test was calculated using the acute stage data of all patients. Both instrumental and vocal music listening activated bilaterally the STG and MTG, as well as the left HG and the right insula and PreCG. The instrumental music also activated the left PreCG and the right HG. In addition to the shared activations, vocal music listening activated bilaterally the IFG, putamen, and cerebellum as well as the left insula, supplementary motor area (SMA), thalamus, hippocampus, and the right cuneus. Vocal > Instrumental condition resulted in activations in the left STG, MTG, and HG. For all the significant results, please see the Table 1 and Fig. 1.

**Table 1:** fMRI activations induced by instrumental and vocal music listening in the acute stage shown by one sample t-tests including all patients.

ACUTE STAGE					
Condition	Fig. 1 panel	Area name	Coordinates	Cluster size	t-value
Instrumental	A	Left Superior Temporal Gyrus (BA 22, 41)	−50 −10 3	3468	7.39**
		Left Middle Temporal Gyrus (BA 21)	−64 −14 −1		
		Left Heschl’s Gyrus (BA 41, 42)	−60 −14 9		
		Left Precentral Gyrus (BA 43)	−54 −12 14		
		Right Superior Temporal Gyrus (BA 22)	52 0 −1	3791	6.76**
		Right Middle Temporal Gyrus (BA 21)	54 −31 −1		
		Right Heschl’s Gyrus (BA 41)	42 −23 9		
		Right Insula (BA 22)	46 −8 −1		
		Right Precentral Gyrus (BA 43)	54 −12 9		
Vocal	B	Left Heschl’s Gyrus (BA 42)	−60 −12 9	19046	8.36**
		Left Superior Temporal Gyrus (BA 22)	−58 −6 1		
		Left Middle Temporal Gyrus (BA 38)	−52 12 −17		
		Left Insula (BA 13)	−42 −8 −5		
		Left Inferior Frontal Gyrus (BA 47)	−42 28 −3		
		Left Putamen	−26 8 1		
		Left Thalamus	−2 −12 13		
		Left Hippocampus (BA 34)	−14 −8 −17		
		Left Cerebellum	−26 −62 −21		
		Right Superior Temporal Gyrus (BA 40, 42, 22)	54 −24 15		
		Right Middle Temporal Gyrus (BA 21)	52 2 −14		
		Right Insula (BA 22)	48 6 −5		
		Right Precentral Gyrus (BA 43)	56 −12 9		
		Right Inferior Frontal Gyrus (BA 47)	42 32 −3		
		Right Putamen	24 16 −1		
		Right Cuneus (BA 17)	18 −82 5		
		Right Cerebellum	24 −58 −21		
		Left Supplementary Motor Area (BA 6)	−46 −6 49	742	4.60*
Vocal > Instrumental	C	Left Superior Temporal Gyrus (BA 22, 41)	−64 −14 −3	914	4.97*
		Left Middle Temporal Gyrus (BA 21)	−64 −22 −1		
		Left Heschl’s Gyrus (BA 42)	−56 −16 10		

*p < 0.05 FWE-corrected at the cluster level. **p < 0.001 FWE-corrected at the cluster level. BA = Brodmann area.

### fMRI activation patterns to music in amusic vs. non-amusic patients

Activation differences comparing non-amusic and amusic patients were evaluated both longitudinally and cross-sectionally. To evaluate longitudinal changes, a flexible factorial analysis of variance (ANOVA) with Group and Time as factors was calculated and six different Group (Non-amusic > Amusic, Amusic > Non-amusic) × (3 months > Acute, 6 months > Acute, 6 months > 3 months) interactions were calculated. Cross-sectional differences were evaluated by calculating two-sample t-tests comparing non-amusic and amusic patients in each time point. Both longitudinal and cross-sectional analyses were done separately for each condition of interest (Vocal, Instrumental, Vocal > Instrumental).

**Figure 2:**
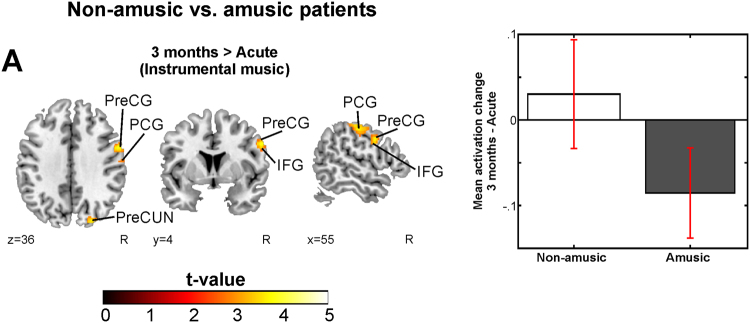
Longitudinal fMRI results during music listening – comparisons between the non-amusic and amusic patients and between the recovered and non-recovered amusic patients. Longitudinal activation pattern changes of (a) non-amusic vs. amusic patients in 3 months > acute (Instrumental); N = 41. Results are shown at *p* < 0.005 (uncorrected) with ≥50 voxels of spatial extent and overlaid over a canonical template with MNI coordinates at the bottom right of each slice (see also Table 2). Only clusters surviving a FWE-corrected *p* < 0.05 threshold are labeled. Bar plot for mean cluster activation change in 3 months – Acute in significant cluster is shown: bar = mean, error-bar = standard error of the mean. IFG = inferior frontal gyrus, PCG = postcentral gyrus, PreCG = precentral gyrus, PreCUN = precuneus.

Longitudinal analyses of the Instrumental condition revealed a significant Group (Non-amusic > Amusic) × Time (3 months > Acute) interaction, with the non-amusic patients showing increasing activation in the right PreCG, PCG, and IFG compared to the amusic patients (Fig. 2, Table 2). No other significant interaction effects were observed.

**Table 2:** Longitudinal fMRI activation increases during music listening in acquired amusia. The other interactions tested yielded no significant results.

3 MONTHS > ACUTE							
Contrast	Fig. 2 panel	Condition	Area name	Coordinates	Cluster size	t-value	R
Non-amusic > Amusic	A	Instrumental	Right Postcentral Gyrus (BA 2, 3)	48 −18 55	776	4.78*	n.s.
			Right Precentral Gyrus (BA 4, 6)	56 2 33			
			Right Inferior Frontal Gyrus (BA 9)	54 5 29			

*p < 0.05 FWE-corrected at the cluster level. R = Pearson correlation (2-tailed p-value, FDR-corrected). The mean activation change in the cluster is correlated to the MBEA average % change over the corresponding time change. BA = Brodmann area, n.s. = not significant.

**Figure 3:**
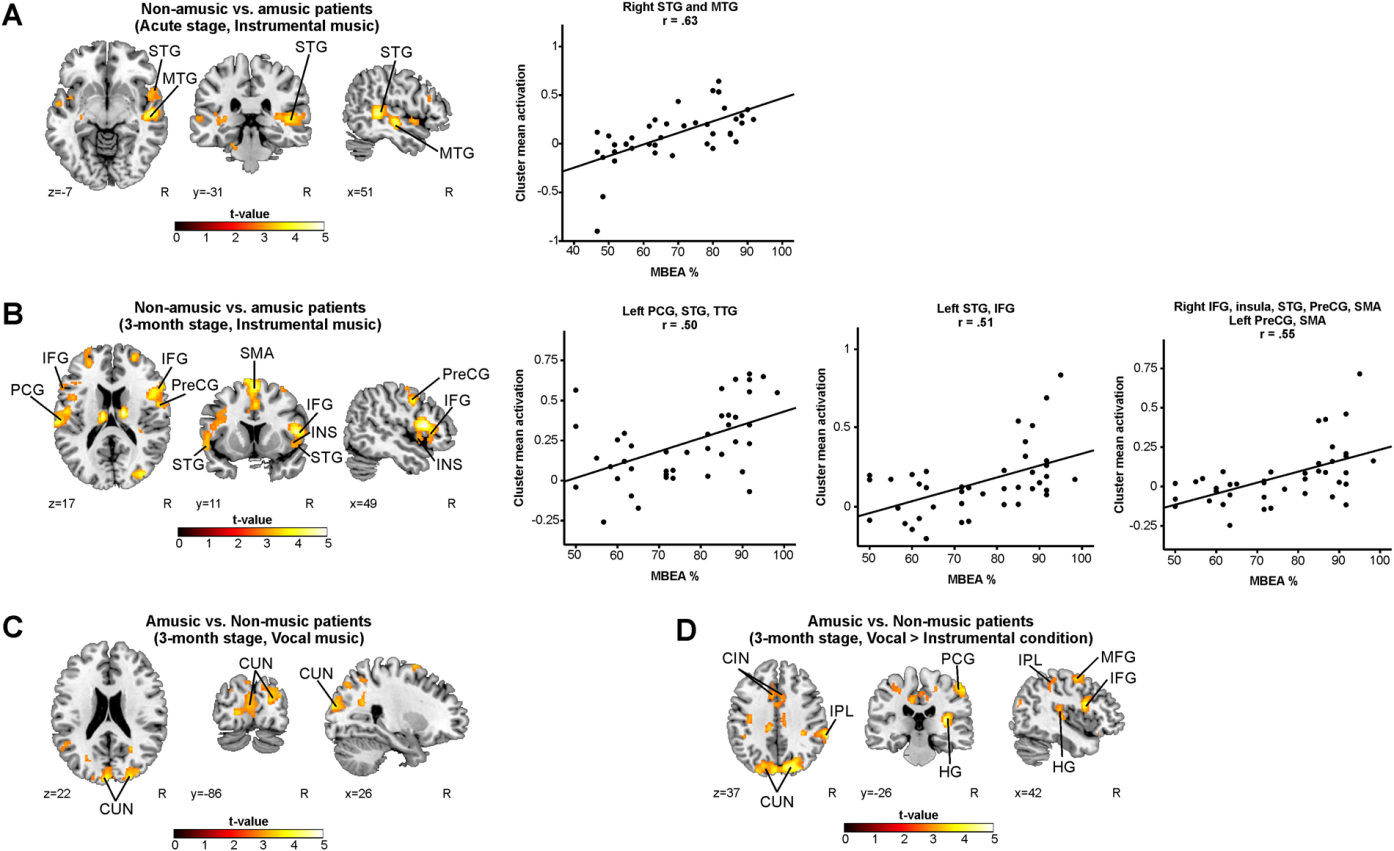
fMRI activation patterns during music listening – a comparison between the non-amusic and amusic patients. Activation patterns of non-amusic vs. amusic patients during music listening tasks showed by t-tests. (**A**) Non-amusic vs. amusic patients (Acute, Instrumental); (**B**) Non-amusic vs. amusic patients (3-month stage, Instrumental); (**C**) Amusic vs. Non-music patients (3-month stage, Vocal); (**D**) Amusic vs. Non-music patients (3-month stage, Vocal vs. Instrumental). N = 41. Results are shown at *p* < 0.005 (uncorrected) with ≥50 voxels of spatial extent and overlaid over a canonical template with MNI coordinates at the bottom right of each slice. Only clusters surviving a FWE-corrected *p* < 0.05 threshold are labeled (see also Table 3). The scatter plots display the correlation between the mean cluster activation and the MBEA total score across the whole sample. CIN = cingulate gyrus, CUN = cuneus, HG = Heschl’s gyrus, IFG = inferior frontal gyrus, INS = insula, IPL = inferior parietal lobule, MFG = middle frontal gyrus, MTG = middle temporal gyrus, PCG = postcentral gyrus, PreCG = precentral gyrus, SMA = supplementary motor area, STG = superior temporal gyrus.

In the cross-sectional analyses of the Instrumental condition, the amusic patients showed significantly reduced activations in the right STG and MTG compared to the non-amusics at the acute stage (Fig. 3A, Table 3). This cluster also correlated strongly with the acute stage MBEA score (R = 0.63, FDR-corrected P < 0.001). At the 3-month stage the defective activation pattern was more wide-spread: the amusics showed less activity bilaterally in the IFG, PreCG, STG, SMA, and in the right insula and cerebellum as well as in the left PCG and HG (Fig. 3B, Table 3). The MBEA performance at 3-month stage correlated significantly with the mean activity observed in the clusters in the right IFG (R = 0.55, FDR-corrected P < 0.001) and in the left PCG (R = 0.50, FDR-corrected P = 0.001) and the STG (R = 0.51, FDR-corrected P = 0.001).

**Table 3:** fMRI results during music listening – a comparison between the non-amusic and amusic patients. The other contrasts tested yielded no significant results.

ACUTE STAGE							R
Contrast	Fig. 3 panel	Condition	Area name	Coordinates	Cluster size	t-value	
Non-amusic > Amusic	A	Instrumental	Right Superior Temporal Gyrus (BA 22)	46 -40 5	1422	5.09*	0.629 (<0.001)
			Right Middle Temporal Gyrus (BA 21)	54 −16 −5			
**3-MONTH STAGE**							
**Contrast**	**Fig. 2 panel**	**Condition**	**Area name**	**Coordinates**	**Cluster size**	**t-value**	**R**
Non-amusic > Amusic	B	Instrumental	Right Inferior Frontal Gyrus (BA 44)	50 16 13	5926	5.22**	0.547 (<0.001)
			Right Insula (BA 22)	48 8 −3			
			Right Superior Temporal Gyrus (BA 38)	54 −16 −7			
			Right Precentral Gyrus (BA 4)	56 −4 17			
			Left Precentral Gyrus (BA 6)	−48 −6 53			
			Right Supplementary Motor Area (BA 6)	2 12 65			
			Left Supplementary Motor Area (BA 6)	−5 −4 69			
			Right Cerebellum	22 −56 −25	596	4.27*	n.s.
			Left Postcentral Gyrus (BA 40, 43)	-64 −22 17	587	4.18*	0.497 (0.001)
			Left Superior Temporal Gyrus (BA 41)	−44 −34 11			
			Left Heschl’s gyrus (BA 42)	−61 −21 12			
			Left Superior Temporal Gyrus (BA 22)	−52 2 3	868	4.13*	0.508 (0.001)
			Left Inferior Frontal Gyrus (BA 44)	−56 16 17			
Amusic > Non-amusic	C	Vocal	Left Cuneus (BA 17)	−16 −82 3	2232	4.39**	n.s.
			Right Cuneus (BA 18)	22 −90 21			
Amusic > Non-amusic	D	Vocal > Instrumental	Right Superior Occipital Gyrus (BA 19)	34 −86 23	9053	5.32**	n.s.
			Right Cuneus (BA 18)	10 −80 25			
			Right Precuneus (BA 5)	4 −42 49			
			Right Cingulate Gyrus (BA 24)	2 2 41			
			Left Cuneus (BA 19)	−6 −92 27			
			Left Supplementary Motor Area (BA 6)	−4 −6 55			
			Left Cingulate Gyrus (BA 32)	−8 12 33			
			Right Inferior Frontal Gyrus (BA 44)	50 10 13	662	4.64*	n.s.
			Right Heschl’s Gyrus (BA 41)	34 −26 11	1734	4.62**	n.s.
			Right Inferior Parietal Lobule (BA 40)	52 −40 51			
			Right Postcentral Gyrus (BA 1)	54 −24 55			
			Right Middle Frontal Gyrus (6)	36 −4 55	955	3.96*	n.s.

*p < 0.05 FWE-corrected at the cluster level. **p < 0.001 FWE-corrected at the cluster level. R = Pearson correlation (2-tailed p-value, FDR-corrected). The mean activation in the cluster is correlated to the MBEA average % of the corresponding point of time. BA = Brodmann area, n.s. = not significant.

Longitudinal analyses of the Vocal condition did not yield any significant interactions. However, in the cross-sectional analyses of the Vocal condition, the amusic patients showed significantly increased activation bilaterally in the cuneus at the 3-month stage compared to the non-amusic patients (Fig. 3C, Table 3). In the Vocal>Instrumental condition, compared to the non-amusic patients, the amusics showed more activation in the right superior occipital gyrus, precuneus, IFG, MFG, HG, inferior parietal lobule (IPL), and PCG as well as bilaterally in the cuneus, cingulate gyrus and in the left SMA at the 3-month stage (Fig. 3D, Table 3). No other significant Group or Group × Time interactions were observed.

### fMRI activation patterns to music in amusia - the effect of aphasia

A subgroup-analysis to evaluate the effect of concurrent aphasia on music activations in amusia was evaluated using the acute stage data. Using Group (Only aphasic/Only amusic/Amusic and aphasic) as a factor, a one-way ANOVA was calculated for each contrast of interest (Vocal, Instrumental, Vocal > Instrumental). Independent t-tests among all groups were calculated for any significant effect.

**Figure 4:**
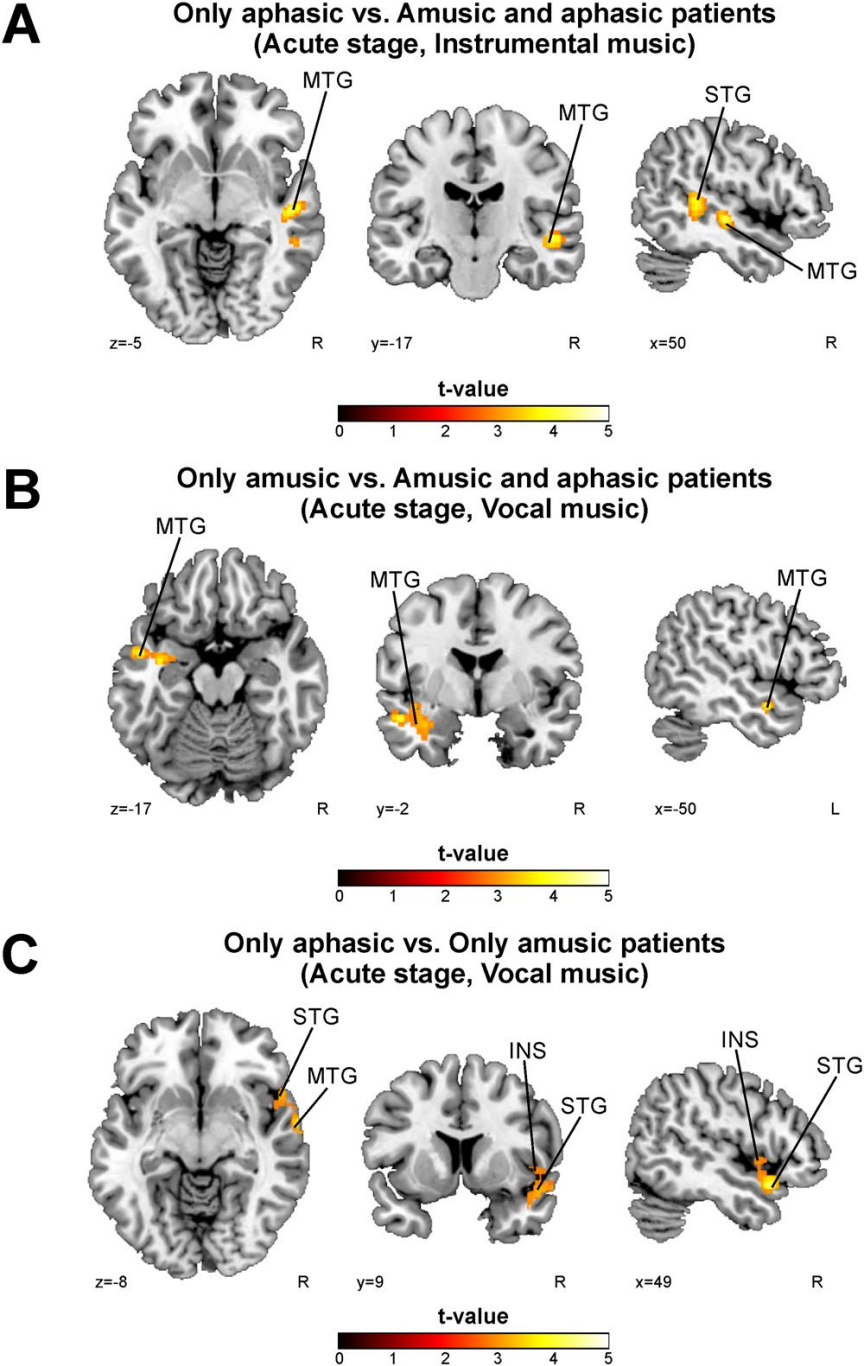
fMRI activation pattern differences between only aphasic, only amusic, and patients with both amusia and aphasia in the acute stage. Results of independent t-tests of exploratory analysis as no cluster survived the correction at the selected threshold. (**A**) Only aphasic vs. Amusic and aphasic patients (Instrumental; p < 0.01 uncorrected at the cluster level); (**B**) Only amusic vs. Amusic and aphasic patients (Vocal; p < 0.05 uncorrected at the cluster level); (**C**) Only aphasic vs. Only amusic patients (Vocal; p < 0.05 uncorrected at the cluster level). N = 23. Results are shown at *p* < 0.005 (uncorrected) with ≥50 voxels of spatial extent and overlaid over a canonical template with MNI coordinates at the bottom right of each slice. See also Table 4. INS = insula, MTG = middle temporal gyrus, STG = superior temporal gyrus.

In the Instrumental condition, patients with only aphasia showed increased activations in the right STG and MTG compared to the patients with both aphasia and amusia (Fig. 4A, Table 4). The mean activity in this cluster also correlated with acute stage MBEA performance (R = 0.69, FDR-corrected P < 0.001). In contrast, in the Vocal condition, patients with only amusia exhibited increased activation of the left MTG compared to the patients with both aphasia and amusia (Fig. 4B, Table 4) In addition, aphasic patients showed increased activations in the right STG, MTG, and insula compared to the amusic patients (Fig. 4C, Table 4). These results were not significant at the corrected cluster level threshold, and therefore should be considered more tentative or exploratory in nature.

**Table 4:** fMRI activation pattern differences between only aphasic, only amusic, and patients with both amusia and aphasia in the acute stage. The other contrasts tested yielded no significant results.

ACUTE STAGE							R
Contrast	Fig. 4 panel	Condition	Area name	Coordinates	Cluster size	t-value	
Aphasic > Amusic&Aphasic	A	Instrumental	Right Superior Temporal Gyrus (BA 21)	44 -38 7	632	4.71**	0.689 (<0.001)
			Right Middle Temporal Gyrus (BA 48)	50 −18 −5			
Amusic > Amusic&Aphasic	B	Vocal	Left Middle Temporal Gyrus (BA 21)	−50 −2 −17	299	4.07*	n.s.
Aphasic > Amusic	C	Vocal	Right Superior Temporal Gyrus (BA 38)	50 12 −17	310	4.32*	n.s.
			Right Middle Temporal Gyrus (BA 21)	64 −4 −11			
			Right Insula (BA 48)	50 6 −3			

*p < 0.05 uncorrected at the cluster level. **p < 0.01 uncorrected at the cluster level. R = Pearson correlation (2-tailed p-value, FDR-corrected). The mean activation in the cluster is correlated to the MBEA average % of the corresponding point of time. BA = Brodmann area, n.s. = non-significant.

### fMRI activation patterns to music in recovered vs. non-recovered amusic patients

First, longitudinal changes were evaluated by calculating a flexible factorial ANOVA with Group and Time as factors, similar to the approach used when comparing non-amusic and amusic subjects. Six different Group (Recovered amusic > Non-recovered amusic, Non-recovered amusic > Recovered amusic) × (3 months > Acute, 6 months > Acute, 6 months > 3 months) interactions were calculated. Again, cross-sectional differences were evaluated by calculating two-sample t-tests separately for each condition of interest (Vocal, Instrumental, Vocal>Instrumental) comparing the two groups (Recovered/Non-recovered) in each time point.

**Figure 5:**
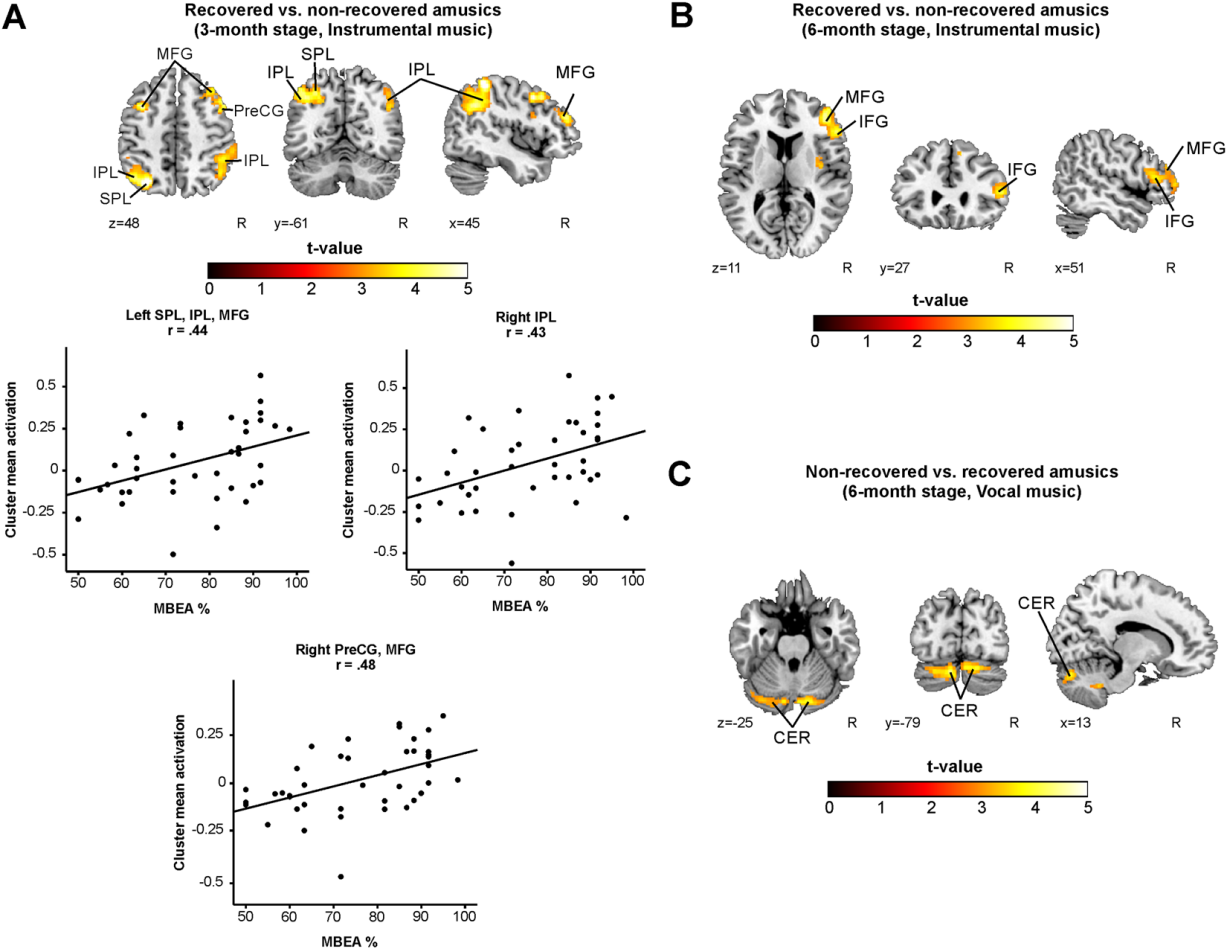
fMRI activation patterns during music listening – a comparison between the recovered and non-recovered amusics. Activation patterns of recovered vs. non-recovered amusic patients during music listening tasks showed by t-tests. (**A**) Recovered vs. non-recovered amusics (3-month stage, Instrumental); (**B**) Recovered vs. non-recovered amusics (6-month stage, Instrumental); (**C**) Non-recovered vs. recovered amusics (6-month stage, Vocal). N = 24. Results are shown at *p* < 0.005 (uncorrected) with ≥50 voxels of spatial extent and overlaid over a canonical template with MNI coordinates at the bottom right of each slice. Only clusters surviving a FWE-corrected *p* < 0.05 threshold are labeled (see also Table 5). The scatter plots display the correlation between the mean cluster activation and the MBEA total score across the whole sample. CER = cerebellum, IFG = inferior frontal gyrus, IPL = inferior parietal lobule, MFG = middle frontal gyrus, PreCG = precentral gyrus, SPL = superior parietal lobule.

Longitudinal analyses of the Instrumental or the Vocal condition did not yield any significant interactions. The significant contrasts between the recovered amusic (RA) and non-recovered amusic (NRA) patients in the cross-sectional analyses are presented in the Table 5 and Fig. 5. In the Instrumental condition, the RAs showed significantly increased activations bilaterally in the MFG and IPL as well as in the left superior parietal lobule (SPL) and the right PCG compared to the NRAs at the 3-month stage (Fig. 5A, Table 5). The MBEA performance in 3-month stage correlated significantly with the mean activity observed in the clusters comprising the right IPL (R = 0.43, FDR-corrected P = 0.005), the right PreCG and MFG (R = 0.48, FDR-corrected P = 0.001) and the left SPL, IPL, and MFG (R = 0.44, FDR-corrected P = 0.004).

**Table 5:** fMRI results during music listening – a comparison between the recovered and non-recovered amusic patients. The other contrasts tested yielded no significant results.

3 MONTHS STAGE							R
Contrast	Fig. 5 panel	Condition	Area name	Coordinates	Cluster size	t-value	
RA > NRA	A	Instrumental	Right Inferior Parietal Lobule (BA 40)	46 −44 57	1480	5.96**	0.430 (0.005)
			Left Superior Parietal Lobule (BA 7)	−30 −68 49	1085	5.86**	0.436 (0.004)
			Left Inferior Parietal Lobule (BA 7)	−36 −66 43			
			Left Middle Frontal Gyrus (BA 9)	−44 −62 49			
			Right Precentral Gyrus (BA 8)	46 8 43	857	5.30*	0.481 (0.001)
			Right Middle Frontal Gyrus (BA 10)	42 42 23			
**6 MONTHS STAGE**							
RA > NRA	B	Instrumental	Right Middle Frontal Gyrus (BA 10)	42 42 13	1304	4.94**	n.s.
			Right Inferior Frontal Gyrus (BA 46)	54 28 11			
NRA > RA	C	Vocal	Left Cerebellum	−12 −70 −37	1101	4.64*	n.s.
			Right Cerebellum	16 −48 −37			

*p < 0.05 FWE-corrected at the cluster level. **p < 0.001 FWE-corrected at the cluster level. R = Pearson correlation (2-tailed p-value, FDR-corrected). The mean activation in the cluster is correlated to the MBEA average % of the corresponding point of time. BA = Brodmann area, NRA = Non-recovered amusic, n.s. = non-significant, RA = Recovered amusic.

At the 6-month stage the RAs showed increased activations in the right IFG and MFG compared to the NRAs (Fig. 5B, Table 5). In the Vocal condition, the NRAs showed significantly increased activation bilaterally in the cerebellum at the 6-month stage (Fig. 5C, Table 5). No other significant Group or Group × Time interactions were observed. None of the correlations between the MBEA performance and cluster mean activity at 6-month stage survived the FDR adjustment. No other significant Group or Group × Time interactions were observed.

### Functional connectivity during music listening in amusia

**Figure 6:**
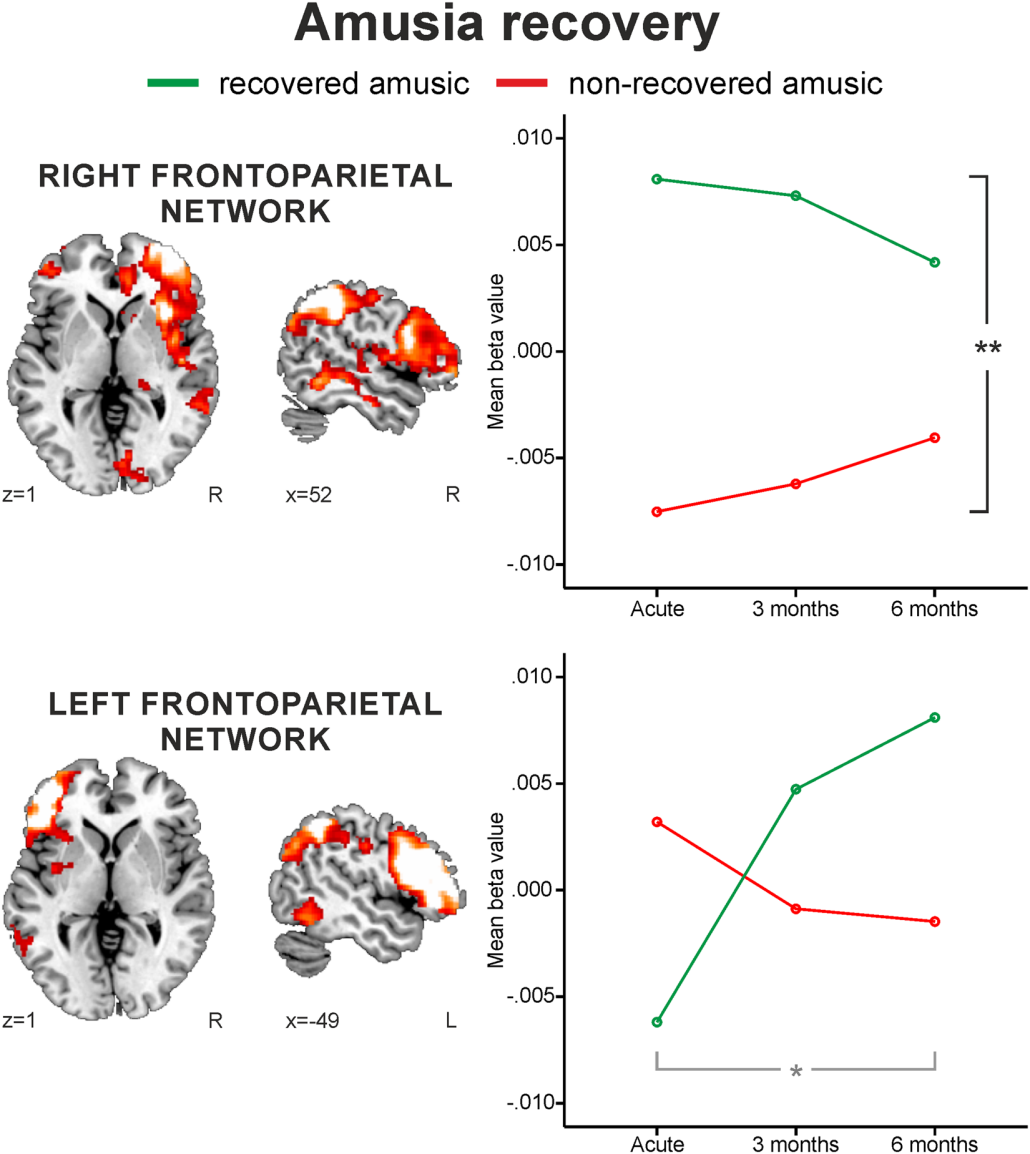
Functional connectivity differences between the recovered and non-recovered amusics during the instrumental music listening. Group (RA/NRA) × Time (Acute, 3 months, 6 months) repeated measures ANOVA results. A representation of the ICA network is shown overlaid over a canonical template with MNI coordinates at the bottom right of each slice. Significant Group effect (black bar), significant Group × Time interaction (gray bar). *p < 0.05 **p < 0.001

The engagement of the four chosen ICA networks (auditory, auditory-motor, left and right frontoparietal) in the Instrumental and Vocal conditions was analyzed using two different mixed-model ANOVAs: Time (Acute/3 months/6 months) and Group (Non-amusic/Amusic) and Time (Acute/3 months/6 months) and Group (RA/NRA). While the first ANOVAs (Non-Amusic/Amusic) yielded no significant effects (minimum P < 0.05), the second ANOVAs (RA/NRA) showed a Group effect for the right frontoparietal network [F (1,22) = 28.20, P < 0.001] and a Time × Group interaction for the left frontoparietal network [F (1.5, 33.39) = 3.93, P = 0.040] in the Instrumental condition (Fig. 6). Compared to the NRAs, the RAs showed greater right frontoparietal network engagement already at the acute stage as well as increasing left frontoparietal engagement from the acute to the 6-month stage. The engagement of the right frontoparietal network correlated significantly with the MBEA score at the 3-month stage (R = 0.46, FDR-corrected P = 0.025).

## Discussion

The present study aimed to uncover functional brain changes associated with acquired amusia after stroke during natural music listening using fMRI and ICA analyses. Our main results show that (i) acquired amusia induced wide-spread dynamic brain activation deficits to instrumental music across time, initiating from right temporal areas at the acute stage, and progressing to bilateral frontal, temporal, and parietal areas at 3 months; (ii) amusia recovery was associated with increased activity to instrumental music in right superior and inferior parietal regions and right inferior frontal areas as well as increased functional connectivity during instrumental music in frontoparietal networks; and (iii) amusic patients showed increased activation to the vocal component of music (Vocal > Instrumental contrast) in left middle temporal areas compared to aphasic patients and in both in many left and right hemisphere areas compared to non-amusic patients. Current results provide important novel information on wide-spread defective processing of music in amusic brain and thus highlight the neural processing patterns that are crucial for music listening.

We have previously shown that acquired amusia after stroke is associated with a right frontotemporal lesion pattern leading to further grey matter volume (GMV) decrease (i.e. atrophy) in the right STG^21, 23^. Overall, there is converging evidence that the right STG is one of the key brain regions in the large-scale network for music processing^4–6^ and plays a crucial role in pitch and melodic processing^18, 53–57^, music syntactic processing^58^, and also in singing^31, 32^. Our present study revealed that acquired amusics exhibited decreased activations to instrumental music in the right STG in the acute and 3-month stage and that the mean activation within these clusters correlated significantly with the MBEA average score. Based on the present results and the previous VLSM and VBM findings, acquired amusia seems to stem from damage to the right temporal region which leads to the observed acute stage activation deficits in the lesioned areas. This is consistent with previous magnetoencephalography results showing that severe acquired amusia is associated with damage to the right AC and consequent decreased function during pitch and duration discrimination processing^59^. On the contrary, congenital amusics exhibit normal activation patterns in the right AC for simple melodic/harmonic stimuli^28, 29^. However, as we did not observe any significant differences between the non-amusics and amusics in the 6-month stage, it is possible that the initial activation deficits in the right superior temporal region normalize over time. In addition, at the 3-month post-stroke stage, the amusic patients showed decreased activations also in the left STG and HG. Previously, congenital amusics have been reported to have abnormal connectivity between the auditory cortices^47^. Thus, it is possible, that the observed left superior temporal activation deficits represent reduced lateral auditory connectivity in acquired amusia.

Previously, congenital amusics have been shown to have dysfunction in the right IFG^44, 45, 47, 60^ and its reduced connectivity to the right AC^28^. The inferior frontal and precentral areas are implicated in sequencing auditory information as well as analyzing the structural relationships and serial prediction in music^58^. Additionally, the IFG is involved in musical priming^61^, recognition of music^62^, perceiving musical emotions^63^, analyzing musical syntax^64^, and performing structural integration of harmonic information^65^. On the grounds of this information, the longitudinal activation decrease in amusic patients compared to non-amusics as well as the cross-sectional activation deficit observed in the right IFG in amusic patients might represent defective processing of music and its components as well as deficient analysis of structural and harmonic information in music. In contrast, compared to the non-recovered amusics, the recovered amusics exhibited compensatory increased activation of the right IFG and MFG during instrumental music listening. Furthermore, as amusic patients showed both cross-sectional and longitudinal activation deficits in the precentral gyrus and cross-sectional activation deficits in the supplementary motor area which are involved in rhythm processing and structural analysis^58, 66, 67^, this further suggests defective higher-order computations in amusia during natural music listening. This might also explain the observed bilateral inferior and superior parietal lobule activation deficits in non-recovered amusics compared to the recovered amusics as the IPL is involved in evaluation of pitch information in tonal structures^68^ as well as in recognition^69^ and deviance detection of melodies^70^ and rhythm perception^70, 71^. The SPL is activated during pitch memory task and musicians have been reported to have increased GMV in the SPL^72, 73^. Together with frontal areas, the parietal regions are also activated during attentive listening to music^74^ and therefore the observed activation deficits in these areas in non-recovered amusics might reflect the inability to focus attention towards natural music and thus impede its neural processing. In addition to the above-mentioned activation deficits, the amusic patients exhibited decreased activation of the right insula and cerebellum. Previously, we have shown that insular lesions are associated with pitch and rhythm processing deficits after stroke^21, 23^. Cerebellum has not been previously implicated in amusia, but it is known to be in involved in processing rhythm^75, 76^ and lyrical content^5^ in music.

One of the key novel findings in our current study was that the acquired amusics showed less activation deficits during listening to vocal music than instrumental music. In addition, we evaluated the effect of language component in music by calculating a Vocal > Instrumental contrast, which revealed that the amusics showed increased activation in the right HG, IFG, MFG, IPL, PCG, superior occipital gyrus, and precuneus as well as in the left SMA and bilaterally in the cuneus and cingulate gyrus compared to the non-amusics. Interestingly, the activation of the right HG, PCG, and IFG has been previously associated with the processing of singing^31, 32^. In addition, the cuneus has been reported to participate in the processing of vocal music^5^ and the precuneus in episodic memory recall^77^. While our current results suggest a less defective processing of vocal music in acquired amusia, the observed increased activation pattern specific to vocal component in music might reflect the amusic brain processing the musical components using the vocal responsive brain regions^5^. Interestingly, congenital amusics can vocalize pitch intervals in correct directions^43^ as well as improve performance in MBEA subtests and song production with singing intervention^41, 42^. This suggests that the processing of vocal music is, at least partially, spared and active in amusic patients. Furthermore, as the congenital amusics can recognize the lyrics of familiar songs while being unable to recognize the corresponding melodies^40^, amusic patients might be able to access the episodic memory through the vocal content of the music rather than via melody. Importantly, as there were no significant differences between the groups (non-amusic vs. amusic/RA vs. NRA) in post-stroke language deficit (i.e. aphasia) occurrence, these differences are unlikely to be specific to language processing dissimilarity between the amusic and the non-amusic patients. At more general level, our results support the dissociation of language and music processing in the brain^33^ as the pattern of defective activations in acquired amusia was greatly different between the instrumental and vocal music.

In addition, we carried out a subgroup analysis comparing patients with amusia without aphasia to patients with aphasia without amusia, or patients with amusia and aphasia. The results of these analyses suggest that areas important for music processing are located in the right temporal and insular areas. In contrast, aphasia affects the processing of vocal music, observed as activation deficits in the left temporal region. While these tentative results need to be interpreted with caution, they support the idea that the disruption in neuronal activity shown by amusic patients during music listening might be more related to music processing deficits rather than to language ones. Furthermore, these results are in line with our previous VLSM findings showing that amusia without aphasia is associated with a lesion pattern comprising the right STG, putamen, and insula, while the lesion pattern in aphasia was localized in the left HG and insula^21^. In addition, lyrics and tunes are processed at varying degrees of integration in superior temporal areas^78^. Our previous VLSM results showed that acquired amusia stems especially from damage to the right hemisphere^21, 23^, and therefore acquired amusia might affect the processing of the instrumental music more than the processing of vocal music. Yet, further studies are needed to provide evidence on the effect of aphasia on amusia.

The cross-sectional results of our current study revealed different patterns of brain activity in acquired amusia during music listening for the different phases after stroke. While the longitudinal analyses were not able to show significant differences between the recovered and non-recovered amusics, comparison between the non-amusics and amusic subjects showed that amusia is associated with decreasing activity during instrumental music listening in the right frontal areas (IFG, PCG, and PreCG). The lack of significant Group × Time interactions in the recovery analysis might be due to the relatively small sample of patients in the groups, especially in the recovered amusic group (N = 9). Another confounding factor could be the longitudinally dynamic brain activations associated with functional recovery, as shown in aphasia^27^. Together with dishinbition^25, 27^ and diaschisis^79^, and the resolution of these two during the post-stroke period, the dynamic shifting of the brain areas might affect the longitudinal analyses. Moreover, in general, the stroke repair-related activity is pronounced in the peri-infarct area, but activity increases also in homologous brain regions in the contralesional hemisphere and in the functionally related network^25^. When evaluating musical deficits after stroke, these above mentioned stroke-related events might hinder longitudinal analyses, especially in evaluation of brain areas engaged in the processing of natural [actual songs as opposed to more simple musical (melodic) sequences] music stimuli, which, in the healthy brain, appear to be bilateral and very wide-spread^4, 5^.

In acquired amusia, the observed differences between the recovered amusics and non-recovered amusics most likely reflect similar longitudinal network reorganization associated with spontaneous recovery of function^25^. Furthermore, in the cross-sectional analyses, the recovery of amusia is associated with increased activity in the right middle and inferior frontal areas as well as bilateral parietal regions. In addition, the amusic patients showed longitudinally decreasing level of activity in the right frontal areas during Instrumental condition compared to non-amusics. Our rationale is further supported by the increased network connectivity observed with ICA in the recovered amusics compared to non-recovered amusics. The recovered amusics had greater right frontotemporal network FC already at the acute stage, indicating that amusia recovery is associated with preserved function and network level connectivity in this network. The engagement of the right frontoparietal network also correlated significantly with the MBEA total score in the 3-month post-stroke stage. In addition, the recovered amusics showed increased engagement of the left frontoparietal network over time reflecting FC changes in the left hemisphere to be associated with amusia recovery. However, while correlations of the increased left frontoparietal FC to MBEA performance did not survive the FDR adjustment for multiple comparisons, the left frontoparietal engagement increase might echo that the recovered amusics regain the access to utilize cross-hemispheric local and global musical networks^8^. Moreover, as the right hemisphere has been suggested to mediate the access to left-lateralized long-term memory representations of music^10^, the initially increased engagement of the right frontoparietal network in recovered amusics might in turn facilitate the longitudinal increase in left side and reflect this maintained/re-established connection to the musical long-term memory. While congenital amusics have been previously shown to have reduced resting-state/intrinsic frontotemporal connectivity^30, 47^, no previous task-ICA analyses exists nor are there studies with acquired amusics on this. Our results provide compelling evidence showing that acquired amusics not only exhibit defective brain activity comprising multiple brain regions during natural music listening, but also show defectively engaging neural networks and that amusia recovery is linked to compensatory network remodeling.

The mechanisms of amusia recovery might be related to spared white matter pathways interconnecting crucial regions underlying music processing. In language research, two processing streams, dorsal and ventral, are widely established to underlie the perception and production of language^80–82^. A similar dual-stream model has been suggested to act parallel in transferring crucial musical auditory information between the temporal, inferior parietal, and inferior frontal regions in the right hemisphere^81, 83–86^. From the two streams, the dorsal (“where” or “how”) pathway connecting the temporal and inferior parietal regions to frontal areas is hypothesized to be important for audio-motor movement and spatial information evaluation, whereas the ventral (“what”) pathway is involved in categorizing sound to auditory objects^81–83, 87^. In aphasia, damage to the dorsal pathway (superior longitudinal and arcuate fasciculus) is associated with productive impairments and, in contrast, comprehension deficits are associated with the ventral pathway (extreme capsule) injury^88^. In the musical domain, it is similarly possible that damage to individual pathways (dorsal or ventral) would manifest in different musical impairments (production vs. perception)^43, 83, 84^. If both the ventral and dorsal pathways are compromised, it is unlikely that the acquired amusia resolves. Instead, patients with at least one preserved right-hemispheric music-related pathway interconnecting frontoparietal regions could engage recovery from acute acquired amusia as the two streams have been found share functionalities and mediate compensatory mechanisms in the language domain^89^. This is supported by our present results, which show that the recovered amusics have greater FC in the right frontoparietal network compared to the non-recovered amusics.

A recent paper reporting exploratory VLSM results suggested that damage to both dorsal and ventral pathways was associated with musical short-term memory deficits^22^. However, it is possible that one of the streams, dorsal or ventral, is more crucial in giving rise to amusia. Previously, the dorsal pathway utilizing auditory-motor conversion circuits has been shown to be involved in vocal stimulus processing^83^. As the amusics did not exhibit decreased activations during vocal music listening compared to non-amusic or recovered amusics in the present study, it is possible that the dorsal pathway is spared, mediating the processing of vocal stimulus in acquired amusia. Moreover, interestingly, congenital amusics have been observed to show dissociations in the functions of dorsal and ventral pathways as they can sing pitch intervals in correct directions while being unable to consciously perceive their differences^43^, as well as improve MBEA performance and song production through singing training^41, 42^ Taken together with the present results, these studies suggest that the dorsal pathway might be spared in amusia and that the crucial white matter neural substrate in amusia is the ventral extreme capsule system. This proposal is in agreement with our previous VLSM results which show that the most critical (highest t-values) acute stroke lesions associated with acquired amusia were located in the right basal ganglia and insular regions, in the extreme capsule^21, 23^. In contrast, preserving the dorsal pathway might facilitate recovery after the acquired amusia, which is reflected by the observed greater FC in the right frontoparietal network in recovered amusics.

While our previously published VLSM results associated acquired amusia strongly with right hemisphere damage^21, 23^, the non-recovered amusics exhibited bilateral activation deficits during instrumental music listening in the present study. One possible explanation is that the critical brain regions in the bilateral music network in the brain are located in the right hemisphere^4–6^ and that damage to these structures manifests in wide-spread processing deficits that lead to decreased fMRI activation during music perception. In this vein, as acquired amusia has been reported also after left hemispheric damage^8^, lesions affecting crucial pathways which interconnect left hemispheric music processing areas to the crucial right hemispheric music-related brain regions (i.e. critical hubs) might lead to acquired amusia. Furthermore, intact right hemisphere has been suggested to compensate for the music perception deficits after left hemisphere damage^8^ thus underlining the importance of the right hemisphere in music processing. One other possible explanation is that as we investigated activation deficits in acquired amusia during full music listening, more global auditory information processing is needed (in contrast to local processing). As shown first by Peretz (1990) and later by Schuppert and colleagues (2000), music perception relies on initial right hemisphere recognition of global musical structures, but it is supported by the left hemisphere subsystems that are dependent on the right hemisphere^8, 90^. Taking these findings together, right hemispheric damage leading to acquired amusia might manifest in wide-spread global music processing deficits whereas left hemispheric damage could affect only local processing and thus lead to small-scale activation deficits. However, while we investigated functional deficits in the current study, research specifically evaluating the white matter connection deficits in acquired amusia have not been published and are therefore needed, especially studies evaluating both processing and perception of music.

As for the limitations of the present study, the possibility of abnormal brain activation during music listening even in non-amusic stroke patients has to be taken into consideration. To overcome this limitation, we evaluated fMRI activations induced by instrumental and vocal music listening as well as the Vocal > Instrumental condition in the acute stage using one sample t-tests including all patients. These resulted in brain activation patterns similar to the previously reported activation patterns during music listening in healthy subjects^4–6^. However, in future studies, an age-matched control group without stroke would help to determine the extent to which the activation patterns to natural music in non-amusic patients are normal (i.e. similar to those of healthy controls). Moreover, while there were no significant differences in the education level or in the pre-stroke musical background information between the recovered and the non-recovered amusic groups, the potential facilitating effect of these variables on amusia recovery needs further investigation.

In conclusion, using naturalistic music listening task, we found that acquired amusics exhibit dysfunction of multiple brain regions previously shown to be a part of the large-scale music network in the brain. The dynamic activation deficits in amusia initiate from the right temporal areas and, over time, proceed to bilateral frontal, temporal, and parietal regions. Most importantly, recovery from acquired amusia is related to increased activation in the right frontal and parietal areas as well as increased functional connectivity in the right and left frontoparietal networks. In addition, amusics showed less activation deficits during listening to vocal music than instrumental music suggesting less defective processing of vocal music. Clinically, these results are important when rehabilitation methods are developed for acquired amusics. Present observations suggest that increasing right as well as left frontoparietal network activity and functional connectivity would be beneficial in the rehabilitation of acquired amusia. Moreover, acquired amusics might benefit from singing-based interventions. Overall, our findings reveal the dynamic nature of acquired amusia after stroke and its neural recovery mechanisms.

## Methods

### Participants

Fifty patients with an acute ischaemic stroke or intracerebral hemorrhage in the left or right hemisphere were recruited between March 2013 and December 2015 from the Division of Clinical Neurosciences of the Turku University Hospital (Tyks). All patients were Finnish-speaking, had normal hearing, were right-handed, under 80 years old, lived in the regions of the Southwest Finland, and were able to co-operate. Patients were enrolled in a larger music listening intervention study. Patients with prior neurological or psychiatric diseases, or drug or alcohol abuse were excluded. All enrolled patients signed an informed consent, and received standard stroke care and rehabilitation. The study was approved by The Ethics Committee of the Hospital District of Southwest Finland, and the study was performed in conformance with the Declaration of Helsinki. Within 3 weeks of the stroke onset, a structural and functional MRI scan and behavioral assessment were performed to all subjects. These were repeated during the follow-up 3 and 6 months post-stroke. Out of the 50 subjects recruited, 41 completed the follow-up and were entered in the final analysis. The clinical and demographic characteristics of the participants are presented in the Table 6.

**Table 6:** Demographic and clinical characteristics of the patients (n = 41).

	Amusia-analysis			Recovery-analysis		
	Amusic n = 24	Non-amusic n=17	*p* value	NRA n = 15	RA n = 9	*p* value
**Demographic**						
Gender (male/female)	16/8	8/9	0.335 (χ2)	8/7	8/1	0.178 (χ2)
Age (years)	58.7 (13.1)	55.4 (14.7)	0.615 (U)	56.9 (14.3)	61 0.8 (10.6)	0.411 (U)
Education (years)	12.3 (3.6)	15.7 (3.8)	0.005 (U)	12.0 (3.3)	12.9 (4.2)	0.770 (U)
**Music background (pre-stroke)**						
Formal music training^a^	0.2 (0.8)	0.7 (1.7)	0.481 (U)	0.0 (0.0)	0.5 (1.4)	0.636 (U)
Other music training^a^	1.0 (2.0)	2.1 (2.2)	0.141 (U)	0.5 (1.5)	1.9 (2.6)	0.325 (U)
Active music listening^b^	4.5 (2.1)	4.8 (2.1)	0.633 (U)	4.5 (2.2)	4.4 (2.0)	1.000 (U)
Passive music listening^b^	6.2 (1.5)	6.3 (1.7)	0.817 (U)	6.4 (1.5)	5.8 (1.6)	0.347 (U)
Musical reward^c^	78.9 (10.2)	73.4 (14.0)	0.233 (U)	79.4 (10.0)	78.0 (11.1)	0.726 (U)
**Clinical**						
Aphasia (no/yes)^d^	8/16	8/9	0.518 (χ2)	7/8	1/8	0.178 (χ2)
MBEA total score% A	58.3 (9.0)	83.7 (4.5)	0.000 (U)	55.7 (9.3)	62.6 (6.8)	0.030 (U)
MBEA total score% 3M	66.8 (11.9)	88.0 (6.1)	0.000 (U)	61.1 (8.9)	76.3 (10.2)	0.001 (U)
MBEA total score% 6M	68.3 (13.2)	88.1 (7.3)	0.000 (U)	61.0 (9.3)	80.4 (9.4)	0.000 (U)
Lesion laterality (left/right)	7/17	13/4	0.004 (χ2)	2/13	5/4	0.061 (χ2)
Lesion volume in cm^3^	67.5 (53.5)	40.4 (47.2)	0.064 (U)	73.7 (58.4)	57.0 (45.2)	0.599 (U)

Data are mean (SD) unless otherwise stated. χ2 = chi-square test, 3M = 3-month stage, 6M = 6-month stage, A = Acute stage, NRA = Non-recovered amusic, MBEA = Montreal Battery of Evaluation of Amusia, RA = Recovered amusic, U = Mann-Whitney U test. ^a^Numbers denote values on a Likert scale where 0 = no, 1 = less than 1 year, 2 = 1–3 years, 3 = 4–6 years, 4 = 7–10 years, and 5 = more than 10 years of training/playing. ^b^Numbers denote values on a Likert scale with a range 0 (does never) to 7 (does daily). ^c^Classification based on Barcelona Music Reward Questionnaire to reflect pre-stroke musical reward. ^d^Classification based on the Boston Diagnostic Aphasia Examination - Aphasia Severity Rating Scale.

### Behavioral assessment

The music perception ability of the patients was evaluated with a shortened version^91^ of the MBEA^92^. Evaluation was carried out in the acute stage (<3 weeks post-stroke) and at the 3-month and 6-month post-stroke stage as a part of a larger neuropsychological testing battery. The stimuli were presented using a laptop and arched headphones.

Following the established cut-off values of the original MBEA^92^ and our previous studies^21, 23, 91^, patients with the MBEA Scale and Rhythm subtest average score <75% at the acute stage were defined as amusic (amusic N = 24, non-amusic N = 17). Based on MBEA scores at the 6-month stage, the amusic patients were further divided into recovered amusics (RA, N = 9), performing above the cut-off, and non-recovered amusics (NRA, N = 15) performing below the cut-off. The MBEA Rhythm and Scale subtest scores correlated strongly at the acute, 3-month, and 6-month post-stroke stages (R(39) = 0.58, 0.67, and 0.69, respectively), and therefore separate subtest analyses were not carried out.

In order to determine whether the activation patterns for music in amusia and its recovery would be similar or different in patients with concurrent aphasia, patients were assessed using the Aphasia Severity Rating Scale (ASRS) from Boston Diagnostic Aphasia Examination (BDAE)^93^. In addition, the performance of the patients in three language tests was used to derive the clinical ASRS estimate: Verbal Fluency Test^94^, shortened Token Test^95^, and shortened Boston Naming test^96^.

### Musical paradigm

A single fMRI session was acquired in all three time points. The patients were presented auditory stimuli consisting of six well-known Finnish songs which were presented to the patients in both full song versions with sung lyrics (vocal music) and in instrumental versions without vocals (instrumental music). A block design with total of 12 blocks of music (six vocal music and six instrumental music blocks) and 12 blocks of rest (no-stimuli) in between the music blocks was used. The duration of each block was 15 seconds. The instrumental music pieces presented were instrumental versions of the vocal music pieces; they were played with various instrumentation (guitar, saxophone, violin) and did not contain any vocal parts (e.g. hummed melody). In the songs, the main melody was sung (vocal music) or played with an instrument (instrumental music). Six different versions of stimuli with randomized order of the blocks were created to overcome the issue of familiarizing to the stimuli during the follow-up sessions. The versions were then randomly ordered to each subject. Musical stimuli were presented through MR-compatible headphones with Presentation software (Neurobehavioral Systems, Inc., Version 16.3 Build 12.20.12).

### MRI data acquisition and preprocessing

The patients were scanned with a 3T Siemens Magnetom Verio scanner using a 12-channel Head Matrix coil (Siemens Medical Solutions, Erlangen, Germany) at the Medical Imaging Centre of Southwest Finland. Functional images were obtained using a single-shot T2*-weighted gradient-echo EPI sequence (slice thickness 3.5 mm; number of slices = 32; TR = 2010 ms; TE = 30 ms; flip angle = 80°; voxel size = 2.8 × 2.8 × 3.5 mm^3^). For the music task, 280 functional volumes were acquired. During fMRI, patients were instructed to lay still with eyes fixed at a fixation point. High-resolution T1-weighted images were acquired for each subject using 3D MPRAGE sequence (slice thickness = 1 mm; number of slices = 176; TR = 2300 ms; TI = 900 ms; TE = 2.98 ms; flip angle = 9°; voxel size = 1.0 × 1.0 × 1.0 mm^3^).

Data were preprocessed using Statistical Parameter Mapping software (SPM8, Wellcome Trust Centre for Neuroimaging, University College, London, UK, www.fil.ion.ucl.ac.uk/spm/) under MATLAB 8.0.0 (The MathWorks Inc., Natick, MA, USA, version R2012b). The fMRI images were initially realigned and a mean image was created. Images were reoriented according to the anterior commissure. Cost function masking was applied to improve the normalization of MRI images with abnormal brain tissue (i.e. stroke patients)^97^. The lesion tracing was done with MRIcron software package (http://people.cas.sc.edu/rorden/mricron/index.html)^98^. Images were then normalized to MNI (Montreal Neurological Institution) space using Unified Segmentation^99^ and re-sampled into 2 × 2 × 2 mm^3^ voxel size. Smoothing was done by using an isotropic spatial filter (FWHM = 8 mm).

The statistical evaluation in each time point (acute, 3 months, 6 months) was based on a least-square estimation using the general linear model. The lesioned areas were included in the fMRI analysis. The different conditions were modeled with a box-car regressor waveform convolved with a canonical hemodynamic response function. Data were high-pass filtered (to a maximum of 1/128 Hz) and serial autocorrelations were estimated using an autoregressive model [AR(1) model]. Confounding factors from head movement were also included in the model. Thus, a block-related design matrix was created including the conditions of interest (Vocal, Instrumental). After model estimation, main effects for both conditions against rest were calculated. In order to detect activation specific to the processing of lyrics, also a direct contrast between the vocal and instrumental conditions (Vocal > Instrumental) was calculated.

### Independent component analysis and calculation of task-related networks

Group Spatial ICA was used to extract the networks present in the fMRI task experiment in the three time points using GIFT software (http://icatb.sourceforge.net/). Following previous studies, the number of possible independent components was set to 20^100–102^. First, the intensity of the acquired images was normalized. Then, using principal component analysis, the data was concatenated and reduced to the 20 temporal dimensions. The data was analyzed using the infomax algorithm^103^. As the intensities of the acquired spatial maps are in percentages of signal change, no scaling was applied. The acquired components were inspected visually to detect deficits and artefacts (e.g. noise). As the right frontotemporal connectivity is thought to be defective in congenital amusia^28, 30, 104^ and music containing lyrics activates frontal and temporal areas bilaterally^5^, we included all components comprising temporal and frontoparietal regions in the final ICA analysis to evaluate the FC changes associated with acquired amusia. Included networks were: (i) auditory, (ii) auditorymotor, and (iii) left and (iv) right frontoparietal (attentional) network.

In order to evaluate which of the four components were associated with the auditory fMRI tasks (i.e. listening to vocal music/instrumental music), three different ICA analyses including all patients were carried out, one for each time point. The ICA spatial components (i.e. networks) were extracted using the whole fMRI run. Then, a time course of each retrieved network in each participant was fitted to an SPM model that included the Instrumental, Vocal, and Rest conditions. Then, beta values representing the engagement of each ICA component were obtained from each condition regressor (i.e. vocal music, instrumental music). This way, the engagement of the selected ICA components was evaluated separately for instrumental music condition and for vocal music condition.

### Statistical analysis

Statistical analyses were carried out using SPM8. First, a one sample t-test including all patients was calculated to evaluate the activation patterns during each condition of interest at the acute stage (Vocal, Instrumental, Vocal > Instrumental, Instrumental > Vocal). To evaluate longitudinal changes, a flexible factorial ANOVA with Group (Non-amusics, RA, NRA) and Time (Acute, 3 months, 6 months) as factors was calculated. To compare RA and NRA groups, six different Group (RA > NRA, NRA > RA) × (3 months > Acute, 6 months > Acute, 6 months > 3 months) interactions were calculated. In addition, to evaluate the longitudinal changes between the non-amusics and amusics, the RA and NRA groups were combined and labelled as amusics, and six different Group (Non-amusic > amusic, Amusic > Non-amusic) × (3 months > Acute, 6 months > Acute, 6 months > 3 months) interactions were calculated. Longitudinal analyses were done separately for each condition of interest (Vocal, Instrumental, Vocal>Instrumental).

Furthermore, as the functional recovery after stroke takes place in three different phases^27^ and that the reorganization includes various elements^25^, also cross-sectional comparisons between the non-amusic vs. amusic and RA vs. NRA groups were calculated. First level contrasts were entered into second-level analyses and two-sample t-tests comparing Non-amusic vs. Amusic or RA vs. NRA were calculated in each time point. This was done separately for each condition of interest (Vocal, Instrumental, Vocal>Instrumental). Additionally, to evaluate the effects of concurrent aphasia on music activations in amusia, a subgroup-analysis of 23 patients [only aphasic (N = 10), only amusic (N = 8), amusic and aphasic (N = 15)] was carried out only in the acute stage (recovery was not assessed due to the small number of patients in this subgroup-analysis). A one-way ANOVA with Group as a factor (Only aphasic/Only amusic/Amusic and aphasic) was used for each contrast of interest. For any significant effect, independent t-tests among all groups were calculated.

Unless otherwise indicated, all results were at a whole-brain uncorrected p < 0.005 at the voxel level. Only clusters surviving a FWE-corrected p < 0.05 at the cluster level with a minimal cluster size set to 50 voxels are reported. Anatomical brain areas were identified using the Automated Anatomical Labelling Atlas^105^ included in the xjView toolbox (http://www.alivelearn.net/xjview/). In addition, Pearson correlations (two-tailed) of the mean activation in individual significant clusters and the MBEA average percentage of the corresponding point of time were calculated. To control for multiple comparisons in cross-sectional correlational analyses, false discovery rate (FDR) approach was used (N = 18), and only significant results are reported (Tables 3–5). Similar approach was applied for the longitudinal analysis comparisons (N = 1).

For the ICA analysis of the fMRI music task, the engagement of the chosen networks during the different listening conditions (Vocal, Instrumental) was evaluated using the extracted beta values. The second level analysis was carried out with SPSS (IBM Corp. Released 2012. IBM SPSS Statistics for Windows, Version 21.0. Armonk, NY: IBM Corp.). Results were analyzed using two mixed-model ANOVAs: [Group (Non-amusic/Amusic) × Time (Acute, 3 months, 6 months)] and [Group (RA/NRA) × Time (Acute, 3 months, 6 months)]. Pearson correlations (two-tailed) of the mean engagement of a significant component and the MBEA average percentage of the corresponding point of time were calculated. FDR approach was used to control for multiple comparisons (N = 6).

As the non-amusic and amusic patients significantly differed in the educational years, a covariate was added for the non-amusic vs. amusic analyses and for the longitudinal analyses. As we have previously shown that persistent acquired amusia after stroke is associated with right hemisphere damage^21, 23^, lesion laterality was not added as a covariate in the analyses. Importantly, the recovered and non-recovered amusics did not differ in any demographic parameters and thus no covariates were used in the amusia recovery analyses.

To verify that the music intervention did not have an effect on amusia recovery, we calculated a mixed-model ANOVA with Time (Acute/3-month/6-month) and Group (3 intervention arms). No significant Time × Group or between-subjects effects were found in the MBEA average score (Time × Group P = 0.772, Group P = 0.339). These results suggest that the music listening intervention did not have any effect on amusia recovery and, therefore, does not impact the results of the present study.

### Data availability

The authors declare that the data supporting the findings of this study are available within the paper.
